# Multimodal neuroimaging and electrophysiological signatures in amnestic mild cognitive impairment with different amyloid-β deposition status: correlations with cognitive function and diagnostic value

**DOI:** 10.3389/fnagi.2026.1896908

**Published:** 2026-09-29

**Authors:** Yang Liang, Peixue Li, Lin Liu, Chunbo Dong, Xiaohong Wang

**Affiliations:** 1 Department of Neuroelectrophysiology, Central Hospital of Dalian University of Technology (Dalian Municipal Central Hospital), Dalian, Liaoning, China; 2 Department of Neurology, The First Affiliated Hospital of Dalian Medical University, Dalian, China; 3 Department of Anesthesiology, Central Hospital of Dalian University of Technology (Dalian Municipal Central Hospital), Dalian, Liaoning, China

**Keywords:** Alzheimer’s disease, amnestic mild cognitive impairment, cognitive function, electroencephalogram, gray matter volume, multimodal biomarker, structural magnetic resonance imaging, β-amyloid

## Abstract

**Background:**

Amnestic mild cognitive impairment (aMCI) is a heterogeneous transitional cognitive state between normal aging and dementia. Aβ positivity elevates dementia conversion risk but does not equate to prodromal Alzheimer’s disease (AD), and not all Aβ-positive aMCI individuals progress to clinical AD dementia. Distinguishing Aβ-positive from Aβ-negative aMCI facilitates pathological subtyping rather than definitive early AD diagnosis. This cross-sectional study aimed to characterize scalp-level resting-state EEG and structural MRI signatures specific to Aβ-positive aMCI, explore their covariate-adjusted correlations with cognitive performance and regional Aβ burden, and develop an internally validated multimodal auxiliary classification model for Aβ stratification.

**Methods:**

Fifty enrolled aMCI participants (30 Aβ-positive, 20 Aβ-negative) and 35 cognitively normal controls (NC) completed unified 20-min eyes-closed resting-state scalp EEG, 3.0 T structural MRI, ^18^F-AV45 amyloid PET and ^18^F-FDG cerebral metabolism PET examinations. Artifact-free scalp EEG spectral power and regional θ/α ratio were calculated via Welch spectral analysis; voxel-based morphometry (VBM) was adopted to quantify whole-brain gray matter volume. Covariate-controlled partial correlation corrected for multiple comparisons was applied to evaluate cross-modal associations among cortical Aβ SUVR load, cerebral glucose metabolism and domain-specific cognitive scores. A binary logistic regression classification model combining screened EEG and MRI markers was established, with DeLong test for ROC curve comparison and 1,000-bootstrap internal validation.

**Results:**

(1) Relative to Aβ-negative aMCI and NC groups, the Aβ-positive subgroup presented significantly elevated whole-scalp θ relative power and scalp θ/α ratio, decreased scalp α and β relative power (all *q* < 0.05, FDR-corrected), with predominant scalp oscillatory alterations distributed over temporal, parietal and occipital scalp electrode clusters. The temporal scalp θ/α ratio yielded optimal classification performance for Aβ stratification (AUC = 0.875). (2) The Aβ-positive subgroup showed significant gray matter atrophy across 13 AD-vulnerable brain regions including bilateral fusiform gyrus, inferior parietal lobule, parahippocampal gyrus, precuneus and right entorhinal cortex (all *q* < 0.05), with the maximum effect size identified in the right precuneus (partial *η*^2^ = 0.477, AUC = 0.778). (3) Within the Aβ-positive subgroup, the temporal scalp θ/α ratio was positively correlated with medial temporal Aβ SUVR (*r* = 0.501, 95% CI [0.146, 0.732], *q* = 0.030); right precuneus gray matter volume was negatively correlated with multi-region cortical Aβ burden (*q* < 0.05). Left parahippocampal gyrus volume was positively correlated with MoCA memory subscores (*r* = 0.479, 95% CI [0.118, 0.709], *q* < 0.05). No significant associations between scalp EEG metrics and FDG glucose metabolism survived FDR correction. (4) The multimodal logistic regression model integrating temporal scalp θ/α ratio and right precuneus gray matter volume achieved favorable classification efficacy for Aβ-positive aMCI differentiation (AUC = 0.852, 95% CI [0.748, 0.955]), with good calibration and clinical net benefit based solely on internal bootstrap validation.

**Conclusion:**

The temporal scalp θ/α ratio and right precuneus gray matter volume are promising candidate non-invasive auxiliary biomarkers for Aβ pathological stratification in aMCI, rather than validated clinical diagnostic biomarkers. The multimodal model provides supplementary subtyping value within this single-center cohort, while external multicenter validation is mandatory before clinical application. This cross-sectional dataset cannot support prediction of longitudinal progression to AD dementia.

## 1 Introduction

Alzheimer’s disease (AD) is the most common neurodegenerative disease characterized by progressive cognitive decline and impaired daily living function, imposing substantial socioeconomic burden on individuals, families and healthcare systems (Livingston et al., 2024). According to standardized global dementia epidemiological reports published by the World Health Organization, approximately 50 million people worldwide live with AD and other dementias, with prevalence projected to rise to 152 million by 2050 (Livingston et al., 2024). Amnestic mild cognitive impairment (aMCI) represents a heterogeneous transitional cognitive stage between unimpaired aging and overt dementia. Aβ positivity only increases the risk of subsequent dementia conversion and cannot be equated to prodromal AD; longitudinal cohort data confirm that only a subset of Aβ-positive aMCI individuals will develop clinical AD dementia over follow-up (Zhou et al., 2024). Existing long-term cohort studies report that 47–75% of aMCI patients exhibit cortical Aβ deposition on PET imaging, yet published conversion rates of 45–82% among Aβ-positive participants cannot be generalized to all aMCI phenotypes. Critically, cross-sectional observational data lack statistical power to predict long-term cognitive deterioration or individual conversion trajectories (Zhou et al., 2024). Importantly, aMCI carries broad pathological heterogeneity: apart from AD-type combined Aβ-tau pathology, cognitive dysfunction can also arise from cerebral small vessel disease, Lewy body pathology and other non-neurodegenerative etiologies (Cardoso et al., 2022). Such mixed pathological backgrounds limit the performance of isolated neuropsychological testing or structural MRI for early pathological subtyping of aMCI, delaying targeted early intervention for individuals at high risk of AD-spectrum progression (Shen et al., 2011).

β-amyloid (Aβ) plaque aggregation constitutes the core pathological hallmark of AD, and ^18^F-labeled Aβ PET remains the clinical reference standard for *in vivo* quantification of cortical amyloid burden (Jack et al., 2013). Pathologically, the spatiotemporal distribution of Aβ and tau lesions exhibits clear divergence at preclinical disease stages: Aβ plaques initially accumulate diffusely across widespread neocortical and default-mode network (DMN) regions, whereas tau neurofibrillary tangles first emerge within medial temporal limbic structures. Medial temporal gray matter atrophy observed in clinical cohorts is primarily driven by tau-mediated neuronal loss rather than primary Aβ toxicity alone (Jack et al., 2013). Stratification of aMCI into Aβ-positive (AD-pathology associated) and Aβ-negative (heterogeneous non-AD) subgroups delivers two core translational benefits: it enables differential diagnosis of mixed cognitive impairment etiologies and supports preliminary risk stratification for cognitive decline trajectory (Liu et al., 2025). Identification of low-cost, repeatable auxiliary biomarkers to distinguish these two subgroups can substantially reduce over-reliance on expensive, radiologically invasive amyloid PET for large-scale preliminary population screening (Liu et al., 2025).

In recent decades, scalp electroencephalogram (EEG) and structural T1-weighted magnetic resonance imaging (sMRI) have attracted widespread translational research interest for early AD-spectrum screening, owing to their non-invasive nature, low cost and broad clinical accessibility. Resting-state closed-eye scalp EEG records synchronized oscillatory potentials generated by large populations of superficial cortical neurons over millisecond time windows. Without source reconstruction algorithms, scalp recordings cannot directly measure deep limbic neuronal activity or single-cell firing events, which avoids overinterpreting surface electrophysiological signals as direct readouts of medial temporal subcortical function. Consistent quantitative spectral shifts, including elevated slow-wave power and attenuated alpha oscillation magnitude, have been repeatedly detected in AD and Aβ-positive aMCI cohorts in prior clinical quantitative EEG research (Rodriguez et al., 2026). Structural MRI combined with voxel-based morphometry (VBM) enables whole-brain quantitative gray matter volume quantification, and AD-related neurodegeneration consistently induces focal atrophy within medial temporal memory circuits and core DMN hubs (Shimoda et al., 2015).

Prior translational research has begun to characterize neuroimaging and electrophysiological phenotypic differences stratified by cortical Aβ burden, yet systematic multimodal joint analyses combining scalp EEG and VBM structural metrics remain scarce in homogeneous pure aMCI cohorts (Park et al., 2025). Two landmark clinical cohorts stratified MCI participants via amyloid PET and identified divergent scalp EEG and regional gray matter signatures between Aβ-positive and negative subgroups, laying foundational evidence for Aβ-stratified biomarker profiling (Nakamura et al., 2018; Tomadesso et al., 2018). Nevertheless, most existing published studies only analyze single-modality imaging or electrophysiological indicators separately, and integrated two-modality predictive pipelines optimized for amyloid subtyping lack sufficient validation in uniform pure amnestic MCI populations (Kim et al., 2021). A recent multimodal analytical framework confirmed complementary diagnostic gain when combining electrophysiological and volumetric biomarkers, yet dedicated validation restricted to pure aMCI cohorts is still absent (Gallego-Rudolf et al., 2025). Beyond auxiliary pathological classification, systematic characterization of subtype-specific multimodal neural signatures improves mechanistic understanding of Aβ-associated neurodegeneration and provides standardized participant stratification reference for future disease-modifying clinical intervention trials (Park et al., 2025). Furthermore, cross-modal associations between scalp EEG metrics, regional gray matter volume, cortical Aβ burden, cerebral glucose metabolism and domain-specific cognitive performance across distinct Aβ subgroups remain insufficiently clarified in existing literature. Single-modal biomarkers inevitably carry inherent limitations for discriminating Aβ-positive aMCI, and the supplementary diagnostic value of integrating scalp EEG and structural MRI requires further targeted verification in homogeneous aMCI cohorts (Kim et al., 2021).

This cross-sectional single-center study systematically integrated resting-state scalp EEG, structural VBM-MRI, ^18^F-AV45 amyloid PET, ^18^F-FDG metabolism PET and standardized neuropsychological assessment to quantify multimodal neural alterations across aMCI subgroups stratified by cortical Aβ deposition status. The predefined research objectives were as follows: (1) Characterize scalp EEG spectral and regional gray matter volumetric phenotypic signatures specific to Aβ-positive aMCI; (2) Quantify covariate-adjusted partial correlations between multimodal neural indicators, regional Aβ SUVR, cerebral glucose metabolism and domain-specific cognitive performance; (3) Screen candidate non-invasive auxiliary biomarkers for preliminary identification of Aβ-positive aMCI; (4) Construct a two-modality logistic regression auxiliary classification model combining scalp EEG and structural MRI metrics to provide a low-cost supplementary pathological subtyping tool within the AD continuum, with no intention to predict longitudinal cognitive progression to clinical dementia.

## 2 Materials and methods

### 2.1 Study subjects

A total of 50 patients diagnosed with amnestic mild cognitive impairment (aMCI) and 35 cognitively normal controls (NC) were recruited from the Department of Neurology between January 2021 and December 2025. This study was approved by the Institutional Review Board of the First Affiliated Hospital of Dalian Medical University (Approval ID: PJ-KS-KY-2025-266). All procedures were conducted in accordance with the Declaration of Helsinki. Written informed consent was obtained from all participants or their legal representatives prior to enrollment.

Inclusion criteria for aMCI group: (1) Meet the diagnostic criteria for aMCI in the 2018 Chinese Guidelines for the Diagnosis and Treatment of Dementia and Cognitive Impairment (Chinese Guidelines Working Group for Diagnosis and Treatment of Dementia and Cognitive Impairment, Cognitive Impairment Committee, Chinese Medical Doctor Association, 2018); (2) Meet the clinical diagnostic criteria for AD-related MCI in the Chinese Expert Consensus on the Diagnosis and Treatment of Alzheimer’s Disease-related Mild Cognitive Impairment 2024 (Dementia and Cognitive Impairment Group, Chinese Medical Association of Neurology, 2024), with neuropsychological impairment characterized by hippocampal amnestic syndrome, hippocampal and entorhinal cortex atrophy on cranial MRI, and other types of MCI excluded; (3) MMSE score ≥24 points; (4) Mild impairment of complex instrumental activities of daily living, but maintaining independent basic activities of daily living, CDR score = 0.5 points; (5) Aged 50–85 years; (6) Right-handed; (7) Complete neuropsychological scale assessment within 2 weeks after clinical diagnosis.

Importantly, this mandatory imaging inclusion criterion may introduce analytical circularity. Medial temporal gray matter atrophy was used as a pre-screening threshold for participant enrollment, while regional gray matter volume was also adopted as a core structural biomarker to distinguish Aβ-positive and Aβ-negative aMCI in subsequent analyses. This dual use of medial temporal atrophy may artificially exaggerate intergroup volumetric differences between subgroups. Meanwhile, this criterion excludes amnestic MCI patients without hippocampal/entorhinal atrophy caused by vascular damage, Lewy body pathology and other non-AD etiologies, which restricts the phenotypic spectrum of our study cohort and limits the external validity of our findings.

Inclusion criteria for NC group: (1) No subjective memory complaints, or objective neuropsychological test results within the normal range despite complaints: MMSE score ≥28 points, CDR score = 0 points; (2) Normal neuroimaging examination; (3) Aged 50–85 years; (4) Right-handed.

Exclusion criteria for all subjects: (1) A history of stroke related to the occurrence or deterioration of cognitive impairment, multiple or extensive cerebral infarction, or severe white matter lesions; (2) Core symptoms of dementia with Lewy bodies, prominent features of frontotemporal dementia or primary progressive aphasia; (3) Other neurological diseases causing memory and cognitive impairment (Parkinson’s disease, Huntington’s disease, normal pressure hydrocephalus, brain tumor, progressive supranuclear palsy, epilepsy, multiple sclerosis, traumatic brain injury with neurological damage, etc.) or non-neurological diseases (Hashimoto’s encephalopathy, general paresis of the insane, anemia, hepatic encephalopathy, uremic encephalopathy, etc.); (4) Definite interictal epileptiform discharges in EEG recordings; (5) Implantation of cardiac pacemaker, arterial clip, artificial heart valve, ear implant or other metal foreign bodies in the body; (6) A previous diagnosis of severe depression, bipolar disorder, schizophrenia and other neuropsychiatric diseases; (7) A history of alcohol abuse, drug abuse or substance addiction in the past 2 years; (8) Severe systemic diseases (severe renal impairment with serum creatinine >265 μmol/L, severe liver disease with AST/ALT more than 3 times the normal value, severe congestive heart failure (NYHA grade 3–4), acute myocardial infarction in the past 6 months, severe pulmonary dysfunction, severe hematological diseases, etc.); (9) Inability to cooperate with cognitive scale assessment or MRI examination; (10) Other conditions deemed unsuitable for the study by the researchers.

All aMCI patients underwent 18F-AV45 PET/MRI examination, and were divided into Aβ-positive group (*n* = 30) and Aβ-negative group (*n* = 20) according to the visual interpretation and quantitative analysis results of 18F-AV45 PET imaging (BAPL score >1 defined as Aβ-positive). The NC group did not undergo 18F-AV45 PET examination and was screened only by inclusion and exclusion criteria.

### 2.2 General demographic data collection

A questionnaire survey was used to record the general demographic data of all subjects, including gender, age, disease course (the time from the onset of cognitive symptoms to the diagnosis of aMCI in aMCI patients), years of education, body mass index (BMI), hypertension, type 2 diabetes, smoking history, drinking history and family history of dementia.

### 2.3 Neuropsychological assessment

All subjects completed a series of neuropsychological scales assessed by two neurologists with the title of attending physician or above. In case of inconsistent results, the assessment was determined by a chief physician with a higher title. The scales included: (1) Mini-Mental State Examination (MMSE); (2) Montreal Cognitive Assessment (MoCA); (3) Activities of Daily Living Scale (ADL); (4) Hamilton Anxiety Scale (HAM-A); (5) Hamilton Depression Scale (HAMD); (6) Hachinski Ischemic Score (HIS). MoCA score was corrected for education years (1 point added for education years ≤12 years).

### 2.4 Resting-state EEG data collection and analysis

#### 2.4.1 EEG data collection

Resting-state closed-eye scalp EEG was recorded in a dedicated quiet electrophysiology laboratory using a Japanese photoelectric digital EEG amplifier system. Pre-test preparation instructions prohibited caffeine, nicotine and alcohol intake on the acquisition day; standardized scalp skin abrasion was performed to reduce electrode-skin impedance below 5 kΩ. Participants remained seated in a dim, sound-attenuated room with eyes closed, maintained awake relaxed vigilance, and continuous 20-min raw EEG recordings were acquired. A 21-channel electrode cap arranged per the international 10–20 system captured signals, amplifier bandwidth 0.1–80 Hz, sampling frequency 1,000 Hz, with bilateral mastoid (A1 + A2) as online reference (Zhang et al., 2019). Add vigilance control details responding to Reviewer 2 Comment 2: Real-time video monitoring was maintained throughout the entire recording session to track participant vigilance, detect eye closure/opening transitions and suppress drowsiness-induced slow-wave artifact contamination.

#### 2.4.2 EEG visual severity grading

Blinded visual grading of background EEG activity was independently completed by two board-certified clinical electroencephalographers, following diagnostic criteria outlined in Clinical Electroencephalography (Liu, 2006); grading categories: normal, borderline, mild abnormal, moderate abnormal, severe abnormal. Inter-rater discrepancies were resolved via joint consensus review.

#### 2.4.3 EEG preprocessing, artifact control and regional partitioning

Raw EEG preprocessing was executed within MATLAB. Sequential preprocessing steps included 1–55 Hz band-pass filtering and 50 Hz mains notch filtering to eliminate power-line interference; common average reference (CAR) re-referencing was applied to all channels (Alarcon et al., 2000). Artifact segments containing blink, saccade and large head-movement contamination were removed via combined visual inspection and semi-automatic sliding-window algorithms (25 s window; segments with >30% artifact contamination discarded). The average valid artifact-free recording duration per participant was 6.2 ± 1.3 min, with minimum valid data proportion ≥30%. Add artifact rejection statistical details responding to Reviewer 2 Comment 2: Total excluded artifact segment duration was calculated for each participant, and one-way ANOVA confirmed no statistically significant differences in artifact rejection rates across NC, Aβ-negative aMCI and Aβ-positive aMCI subgroups (*q* > 0.05). All participants exhibited sustained awake vigilance per synchronized video monitoring, with no extended drowsiness episodes recorded during acquisition. Post-processing data quality visual inspection was performed channel-by-channel to eliminate residual noisy traces prior to spectral decomposition. For regional scalp electrode partitioning, the 21 recording channels were grouped into five scalp surface anatomical clusters: frontal (FP1, FP2, F3, Fz, F4), central (C3, Cz, C4), temporal (T3, T4, T5, T6, F7, F8), parietal (P3, Cz, P4), occipital (O1, O2) (Daly et al., 2013). Add explanation for overlapping Cz electrode responding to Reviewer 2 Comment 7: Overlap of electrode Cz across central and parietal scalp clusters creates minor partial collinearity between central and parietal spectral metrics; this partitioning scheme was retained to align with conventional clinical EEG regional reporting frameworks, and this overlapping electrode limitation is acknowledged in the Discussion section. Continuous artifact-free traces were segmented into non-overlapping 5-s epochs for frequency-domain analysis (Daly et al., 2013; McKay et al., 2025).

#### 2.4.4 Spectral decomposition, relative power calculation and normalization

Welch’s periodogram method was utilized for quantitative spectral power estimation (Zhang et al., 2024). Raw signals were downsampled to 250 Hz for computational efficiency; each 5-s epoch (1,250 data points) applied a 125-point Hamming window with 50% inter-window overlap, and 1,024-point fast Fourier transform (FFT) generated frequency resolution ~0.25 Hz. Standard frequency band partitions: δ (1.0–4.0 Hz), θ (4.0–8.0 Hz), α (8.0–13.0 Hz), β (13.0–30.0 Hz). Band-specific relative power values were averaged across all valid epochs and within-cluster electrodes to yield a single representative value per participant per scalp region. Add rationale for min-max normalization responding to Reviewer 2 Comment 8: Min-max linear scaling was applied to normalize relative power values to a standardized [−1, 1] range across all participants. This normalization strategy eliminates inter-individual variation in total absolute spectral power, enabling direct comparison of band oscillation balance independent of global cortical activation magnitude. This transformation is not universally adopted in clinical EEG literature and restricts direct cross-study effect-size comparison with unnormalized spectral metrics, a limitation explicitly addressed in the Discussion section. The scalp θ/α ratio was calculated as the quotient of normalized θ relative power divided by normalized α relative power for each scalp region. Critical interpretative limitation statement responding to Reviewer 1 Major 4: All derived EEG metrics exclusively reflect aggregated oscillatory activity recorded from superficial scalp electrodes overlying cortical gray matter. No source reconstruction or dipole localization analyses were performed, so all EEG-related results are reported strictly at the scalp electrode cluster level; no direct causal inference regarding deep medial temporal subcortical neuronal function is drawn from surface electrophysiological data within the manuscript (Rodriguez et al., 2026; Papp et al., 2017).

### 2.5 PET/MRI examination and analysis

#### 2.5.1 Examination equipment and imaging protocol

All aMCI patients underwent Biograph mMR PET/MRI scanner (Siemens, Germany) for simultaneous PET and MRI image acquisition, including 18F-FDG PET and 18F-AV45 PET imaging to assess brain glucose metabolism and Aβ load, respectively (Yan et al., 2020). For 18F-FDG PET examination, patients maintained a light diet and fasted for 4–6 h, suspended drugs affecting cognitive function and brain metabolism on the examination day, with fasting blood glucose <7.8 mmol/L, and were injected with the tracer at a dose of 0.15 mCi/kg intravenously after resting for 60 min in a quiet and dark environment. For 18F-AV45 PET imaging, 7–10 mCi of the tracer was injected intravenously, and a 25-min static emission scan was performed after a 50-min uptake period. Images were reconstructed iteratively using the time-of-flight ordered subset expectation maximization (TOF-OSEM) algorithm (5 iterations, 20 subsets), with a reconstructed image matrix of 256 × 256, a field of view of 350 × 350 mm2, and a voxel size of 1.2 × 1.2 × 1.4 mm3.

#### 2.5.2 Image processing, analysis and ROI selection

^18^F-AV45 PET and ^18^F-FDG PET images underwent blinded visual assessment and standardized quantitative post-processing. For ^18^F-AV45 amyloid PET, three independent board-certified nuclear medicine physicians performed visual reading without access to clinical, cognitive or structural MRI data. Scans were graded using the Brain Amyloid Plaque Load (BAPL) visual scoring system: 1 = no cortical amyloid uptake; 2 = mild diffuse cortical uptake; 3 = severe widespread cortical uptake. Discrepant scores were resolved via joint consensus discussion, with a BAPL score > 1 defined as the threshold for Aβ positivity. Rationale for combined visual-quantitative classification (addressing Reviewer 1 Minor 10): Dual visual BAPL grading paired with continuous SUVR quantification was adopted rather than a fixed single SUVR cutoff for three reasons: (1) Visual BAPL scoring aligns with routine clinical amyloid PET reporting workflows in domestic neurology practice; (2) No population-specific normative SUVR threshold dataset was available for this single-center cohort; (3) Combined adjudication reduces classification bias introduced by scanner variability and inter-individual tracer uptake differences. For quantitative analysis, standardized uptake value ratio (SUVR) values were calculated for AD-vulnerable cortical ROIs (frontal lobe, anterior cingulate gyrus, lateral parietal lobe, lateral temporal lobe), with cerebellar gray matter as the reference tissue. Aβ-positive and Aβ-negative subgroups were stratified based on integrated visual BAPL grading and global cortical SUVR metrics.

^18^F-FDG PET images were processed quantitatively using FreeSurfer v7.4. First, co-registered T1-weighted structural MRI images underwent automated tissue segmentation and anatomical labeling: subcortical nuclei were segmented using the aseg atlas, while cortical surfaces were parcellated according to the Desikan-Killiany (DK) atlas, yielding 34 distinct cortical regions and 9 subcortical structures per cerebral hemisphere. All segmentation outputs underwent case-by-case visual quality control; manual editing corrected obvious segmentation misalignment, and scans with uncorrectable severe segmentation artifacts were excluded from FDG analysis. Each ^18^F-FDG PET volume was rigidly co-registered to its corresponding anatomical segmentation space, and mean raw SUV values were extracted per atlas-defined ROI. To eliminate inter-individual variation in global cerebral tracer uptake, signal normalization used pontine gray matter as the reference region to generate FDG-SUVR. Volume-weighted average FDG-SUVR values from homologous left and right hemisphere ROIs were computed for subsequent statistical testing (Kim et al., 2024; Lee et al., 2024). Supplementary statement for Reviewer 1 Minor 7: All regional FDG-SUVR values were statistically compared between Aβ-positive and Aβ-negative aMCI subgroups, yet no significant intergroup metabolic differences survived FDR multiple comparison correction. Accordingly, FDG metabolic metrics were not integrated into the primary multimodal classification model for Aβ stratification, and full FDG intergroup comparison results are presented in the Results section for transparency.

AD-pathology vulnerable composite ROIs for cross-modal partial correlation analyses were predefined as follows: (1) posterior cingulate/precuneus complex; (2) lateral parietal association cortex; (3) lateral temporal cortex; (4) medial temporal lobe composite (hippocampus + parahippocampus + entorhinal cortex); (5) medial prefrontal cortex; (6) global whole-cortex average SUVR.

### 2.6 Structural MRI data collection and VBM analysis

#### 2.6.1 MRI data collection

All participants completed 3.0 T structural MRI scanning on a uMR Omega scanner (United Imaging Healthcare, China) equipped with a 32-channel head coil (Aydın et al., 2017). Participants lay supine with head immobilized, eyes closed and maintained wakefulness throughout acquisition. 3D T1-weighted anatomical sequence parameters: repetition time (TR) = 8.1 ms; echo time (TE) = 3.1 ms; flip angle = 9°; field of view (FOV) = 300 × 300 mm^2^; matrix size = 256 × 256; contiguous 1 mm axial slices; native voxel dimension = 1.17 × 1.17 × 1 mm^3^; total of 180 slices acquired (Sun et al., 2020).

#### 2.6.2 VBM analysis and gray matter volume extraction

Voxel-based morphometry (VBM) analysis was performed using the FreeSurfer software package (version 7.4) for automatic brain image segmentation and volume quantitative analysis (Wierenga et al., 2014). The processing flow included intensity standardization, bias field correction, non-brain tissue removal, Talairach space transformation and other preprocessing steps; then, based on the tissue probability atlas and surface modeling technology, the cortical and subcortical gray matter, white matter and cerebrospinal fluid were accurately segmented, and multiple brain anatomical regions were automatically labeled. Finally, the gray matter volume data of each specific brain region of each subject were extracted from the processing results for subsequent statistical comparison. Based on the automatic segmentation and labeling results of the Desikan-Killiany cortical parcellation atlas built in FreeSurfer, the gray matter volume data of a total of 68 ROIs in the bilateral cerebral hemispheres of each subject were systematically extracted (Sarazin et al., 2010). All extracted ROI gray matter volume data were subjected to normality test and outlier processing.

### 2.7 Statistical analysis

SPSS 27.0 software and R software (version 4.3.0) were used for statistical analysis. The measurement data were expressed as mean ± standard deviation (x ± s), and the count data were expressed as frequency (percentage). For the comparison of baseline data among the three groups (Aβ-positive, Aβ-negative, NC), one-way analysis of variance (ANOVA) was used for measurement data, and Bonferroni correction was used for *post hoc* pairwise comparison; the *χ*^2^ test was used for count data, with continuity correction for 1 ≤ theoretical frequency <5, and Fisher’s exact probability method for theoretical frequency < 1.

For the comparison of EEG relative power among the three groups, analysis of covariance (ANCOVA) was used with age, gender and years of education as covariates. The Shapiro–Wilk normality test was performed on the ANCOVA residuals of each indicator, and the Levene test for homogeneity of variance was performed on the original data of each group. If the parametric assumptions of ANCOVA were met, the group was used as the fixed factor, and age, gender and years of education were included as covariates in the analysis. If the main effect of ANCOVA was statistically significant (*q* < 0.05), *post hoc* pairwise comparison was further performed, and the Bonferroni correction method was used to adjust the significance level to control the false positive risk caused by multiple comparisons.

For the comparison of gray matter volume between Aβ-positive and Aβ-negative groups, ANCOVA was used with age, gender and estimated total intracranial volume (eTIV) as covariates. The false discovery rate (FDR) correction was performed on all results to control multiple comparisons, with *q* < 0.05 considered statistically significant.

Pearson or Spearman correlation analysis was used to analyze the correlations between EEG indicators, gray matter volume and MMSE score, MoCA score, Aβ load (SUVR), brain glucose metabolism (FDG-SUVR). Partial correlation analysis was used to control the influence of confounding factors such as age, gender, years of education and eTIV. The *p* values of correlation analysis were all corrected by FDR, with *q* < 0.05 considered statistically significant.

Receiver operating characteristic (ROC) curve analysis was used to evaluate the diagnostic efficiency of EEG indicators and gray matter volume for distinguishing Aβ-positive from Aβ-negative aMCI, and the area under the curve (AUC), sensitivity, specificity, cut-off value and 95% confidence interval (CI) were calculated.

Binary Logistic regression analysis was used to construct a multimodal predictive model for Aβ-positive aMCI. First, single-variable analysis (t test or Mann–Whitney U test according to data distribution) was used to screen EEG indicators and gray matter volume with significant differences between Aβ-positive and Aβ-negative groups, which were standardized (Z score) and included in the binary Logistic regression model. Bootstrap resampling (1,000 times) was used for internal validation to calculate the corrected regression coefficient (β), odds ratio (OR) and 95% CI. Based on the Logistic regression model, the predicted probability value of Aβ-positive status for each subject was saved, and ROC curve analysis was performed to evaluate the comprehensive diagnostic efficiency of the model. Calibration curve combined with Hosmer-Lemeshow test was used to evaluate the consistency between the predicted probability and the actual Aβ status. Decision curve analysis (DCA) was used to evaluate the clinical net benefit of the model at different threshold probabilities. A clinical nomogram was constructed using the rms package of R software to realize the visualization of individualized Aβ-positive risk prediction results, and the C-index and 95% CI were calculated by Bootstrap method (1,000 resampling) to evaluate the prediction accuracy of the nomogram. All statistical tests were two-sided, with *p* < 0.05 or *q* < 0.05 considered statistically significant.

## 3 Results

### 3.1 Comparison of baseline data among three groups

There were no significant differences in age, gender, disease course, years of education, BMI, hypertension, type 2 diabetes, smoking history, drinking history, family history of dementia, ADL, HAM-A, HAMD and HIS scores among the Aβ-positive group, Aβ-negative group and NC group(all raw *p* < 0.05, Table 1). *Post hoc* test showed that the MMSE and MoCA scores of the Aβ-positive group and Aβ-negative group were significantly lower than those of the NC group (all raw *p* < 0.05), and there were no significant differences in MMSE and MoCA scores between the Aβ-positive group and Aβ-negative group (both raw *p* > 0.05, Tables 1, 2).

**Table 1:** Demographic and clinical characteristics and clinical data of participants.

	Aβ-positive (*n* = 30)	Aβ-negative (*n* = 20)	NC (*n* = 35)	Raw *p* value
Age (year)	62.80 ± 7.03	62.50 ± 5.38	62.77 ± 9.10	0.988
Male (*n*, %)	11 (36.7%)	11 (55.0%)	16 (45.7%)	0.437
Disease duration (year)	2.93 ± 1.72	3.90 ± 2.27	—	0.093
Education (year)	11.70 ± 3.02	11.64 ± 3.09	11.77 ± 3.28	0.857
BMI (kg/m^2^)	24.37 ± 2.66	24.87 ± 2.16	24.95 ± 2.27	0.599
Hypertension (%)	6 (20.0%)	6 (30.0%)	12 (34.3%)	0.820
Diabetes (*n*, %)	12 (40.0%)	5 (25.0%)	19 (54.3%)	0.580
Smoking (*n*,%)	4 (13.3%)	4 (20.0%)	6 (17.1%)	0.816
Drinking (*n*, %)	2 (6.7%)	6 (30.0%)	9 (25.7%)	0.127
Family history of dementia (*n*, %)	6 (20.0%)	3 (15.0%)	1 (2.9%)	0.089
MMSE	26.13 ± 2.68	26.95 ± 1.79	28.03 ± 1.12	<0.001*
MoCA	24.33 ± 3.04	24.85 ± 2.83	26.43 ± 1.69	0.030*
ADL	73.65 ± 3.05	73.64 ± 2.76	73.09 ± 2.13	0.230
HAMA	2.87 ± 2.21	2.80 ± 1.74	3.17 ± 2.17	0.769
HDMA	5.60 ± 4.18	3.45 ± 2.52	4.43 ± 3.24	0.098
HIS	0.73 ± 0.69	0.85 ± 0.67	0.71 ± 0.67	0.760

*Raw *p* < 0.05 indicates a statistically significant difference.

**Table 2:** *Post-hoc* comparison of MMSE and MoCA scores among the three groups (raw *p* values).

	Aβ-positive vs. Aβ-negative	Aβ-positive vs. NC	Aβ-negative vs. NC
MMSE	0.492	0.003*	0.044*
MoCA	0.905	0.005*	0.041*

*Raw *p <* 0.05 indicates a statistically significant difference.

### 3.2 Comparison of EEG severity grading among three groups

There were no significant differences in the clinical diagnosis of EEG severity grading among the Aβ-positive group, Aβ-negative group and NC group (all raw *p* > 0.05, Table 3). No moderate or severe abnormal EEG was found in any of the three groups.

**Table 3:** Comparison of EEG severity grading among Aβ-positive, Aβ-negative, and NC groups.

EEG severity grading	Aβ-positive (*n* = 30)	Aβ-negative (*n* = 20)	NC (*n* = 35)	Raw *p* value
Normal EEG	8 (26.7%)	9 (45.0%)	13 (37.2%)	0.396
Borderline EEG	15 (50.0%)	5 (25.0%)	18 (51.4%)	0.127
Mildly abnormal EEG	7 (23.3%)	6 (30.0%)	4 (11.4%)	0.216
Moderately abnormal EEG	0 (0.0%)	0 (0.0%)	0 (0.0%)	0 (0.0%)
Severely abnormal EEG	0 (0.0%)	0 (0.0%)	0 (0.0%)	0 (0.0%)

### 3.3 Comparison of EEG spectral characteristics across groups with different Aβ status

#### 3.3.1 Global relative power

ANCOVA of global normalized scalp EEG relative power identified significant omnibus intergroup effects (Figure 1A). *Post-hoc* pairwise contrasts confirmed that the Aβ-positive aMCI subgroup exhibited significantly elevated theta relative power and theta/alpha ratio, alongside reduced alpha and beta relative power relative to both the Aβ-negative aMCI subgroup and NC cohort (all *q* < 0.05). No statistically significant differences in delta, theta, alpha, beta relative power or global theta/alpha ratio were detected between the Aβ-negative aMCI subgroup and NC participants (all *q* > 0.05).

**Figure 1:**
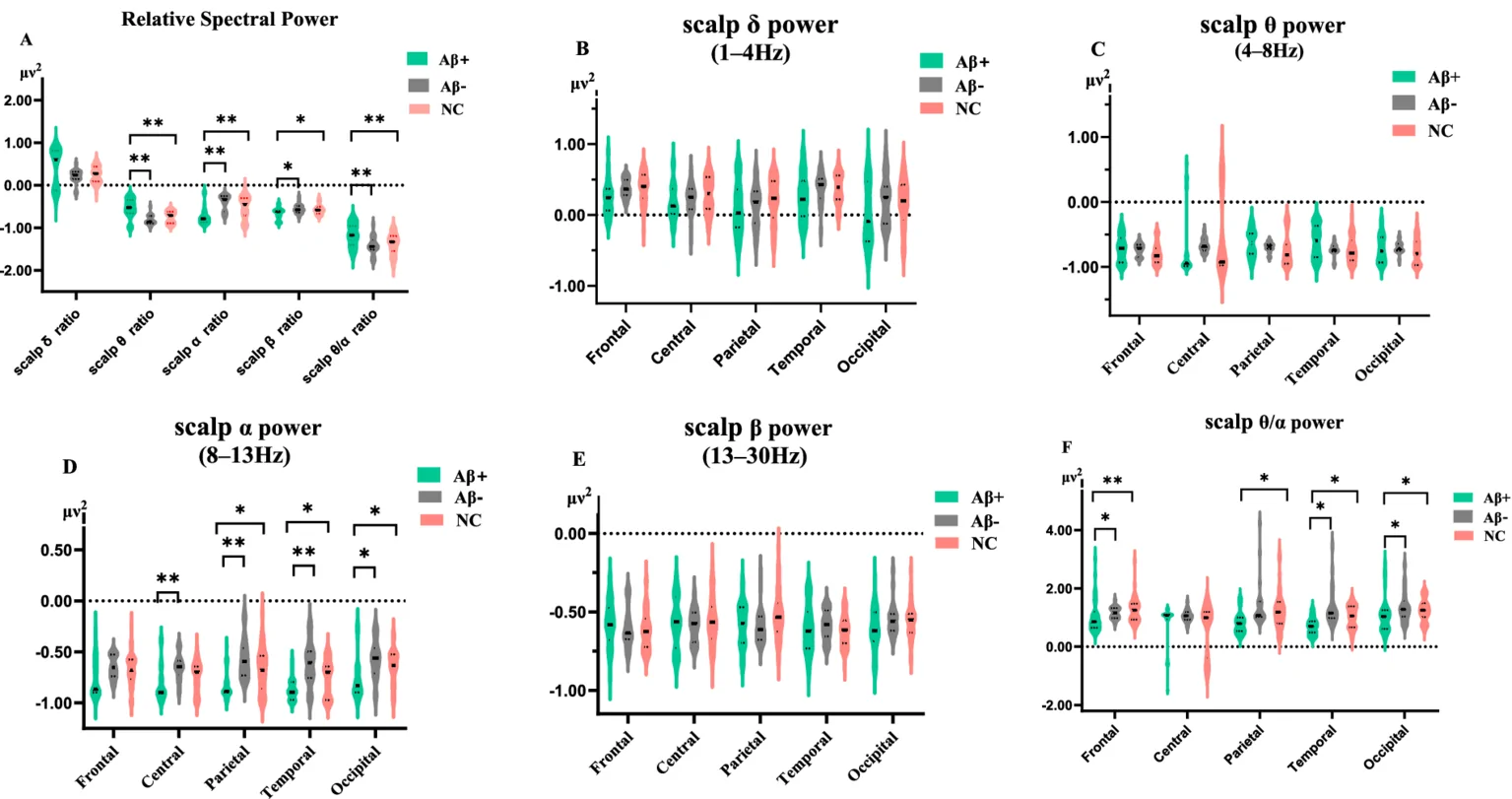
Resting-state scalp EEG spectral power metrics for Aβ-positive aMCI, Aβ-negative aMCI and NC groups. **(A)** Global normalized relative power of δ, θ, α, β frequency bands and global scalp θ/α ratio across three subgroups. **(B–E)** Regional normalized relative power of δ **(B)**, θ **(C)**, α **(D)**, β **(E)** bands calculated within five predefined scalp electrode clusters (frontal, central, parietal, temporal, occipital). **(F)** Regional scalp θ/α ratio across the five scalp partitions. Data are presented as mean ± standard deviation (SD). All statistical contrasts were performed via ANCOVA with Bonferroni *post-hoc* testing; all significance markers correspond to FDR-adjusted q-values. *q < 0.05, **q < 0.01.

#### 3.3.2 Regional relative power

ANCOVA of normalized scalp regional relative power revealed significant omnibus intergroup effects for alpha band relative power and regional scalp θ/α ratio. *Post-hoc* pairwise comparisons demonstrated that the Aβ-positive aMCI subgroup showed significantly decreased alpha relative power within central, parietal, temporal and occipital scalp clusters, together with elevated regional θ/α ratio in frontal, parietal, temporal and occipital scalp clusters relative to both Aβ-negative aMCI and NC cohorts (all *q* < 0.05). Full spatial distribution of these intergroup spectral alterations is visualized in Figures 1B–G.

#### 3.3.3 Diagnostic performance: ROC curve analysis

The ROC analysis showed that normalized delta band scalp relative power only yielded significant discriminative capacity within the frontal scalp cluster (AUC = 0.72, *q* = 0.045), whereas no statistically significant classification performance was detected for delta power in central, parietal, temporal and occipital scalp partitions (all *q* > 0.05).

Normalized alpha band scalp relative power achieved significant differentiation between Aβ-positive and Aβ-negative aMCI subgroups across all five predefined scalp electrode clusters (frontal, central, parietal, temporal, occipital; all *q* < 0.05), with the optimal discriminative performance localized to the temporal scalp region (AUC = 0.86, *q* = 0.005).

The scalp theta/alpha ratio delivered meaningful discriminatory efficacy in four scalp clusters (frontal, parietal, temporal, occipital; all *q* < 0.05), with the central scalp region as the only non-significant partition. This ratio achieved strong classification performance in both parietal (AUC = 0.84, *q* < 0.001) and temporal scalp clusters (AUC = 0.88, *q* < 0.001).

Neither theta nor beta normalized scalp relative power demonstrated significant subgroup discrimination in any scalp region after FDR multiple comparison correction (all *q* > 0.05) (Figure 2).

**Figure 2:**
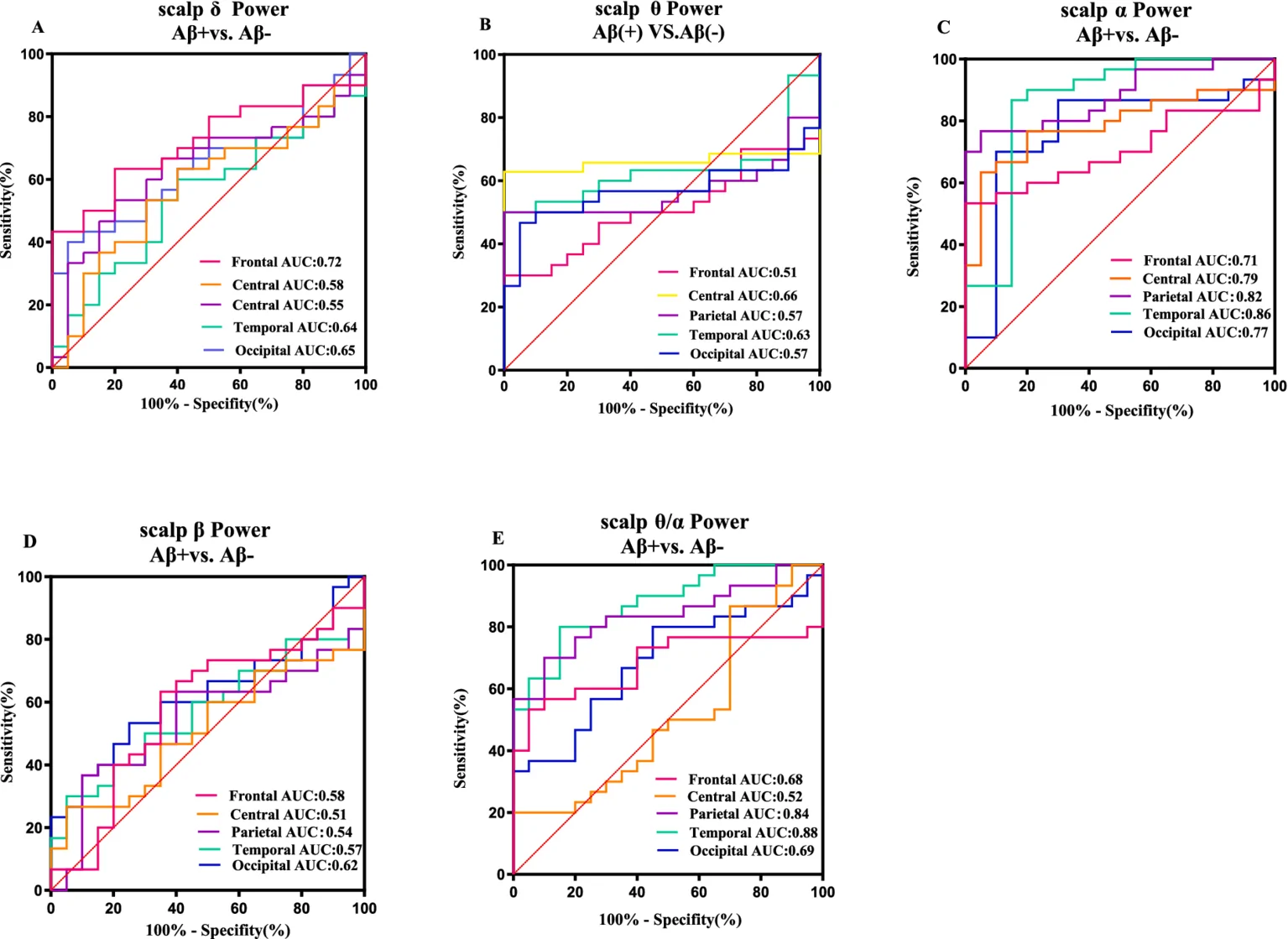
ROC curve analysis of scalp EEG spectral indicators for distinguishing Aβ-positive from Aβ-negative aMCI **(A–E)** Receiver operating characteristic (ROC) curves of regional scalp relative power in δ **(A)**, θ **(B)**, α **(C)**, and β **(D)** bands, as well as the scalp θ/α ratio **(E)**, for differentiating Aβ-positive aMCI from Aβ-negative aMCI. The area under the curve (AUC) values for each scalp electrode cluster are provided in the legend.

### 3.4 Comparison of gray matter volume between Aβ-positive and Aβ-negative groups

Gray matter volume values extracted from 68 FreeSurfer-defined regions of interest (ROIs) were compared between the Aβ-positive and Aβ-negative aMCI subgroups via analysis of covariance (ANCOVA), adjusting for age, sex, and estimated total intracranial volume (eTIV) as nuisance covariates. Statistically significant intergroup volumetric differences (FDR-adjusted *q* < 0.05) were detected across multiple AD-vulnerable cortical and subcortical ROIs. In all significant regions, the Aβ-positive subgroup exhibited reduced gray matter volume relative to the Aβ-negative subgroup. These significant ROIs included left (L.) fusiform gyrus, left (L.) inferior parietal lobule, left (L.) inferior temporal gyrus, left (L.) parahippocampal gyrus, left (L.) posterior cingulate cortex, left (L.) precuneus, right (R.) caudal middle frontal gyrus, right (R.) entorhinal cortex, right (R.) fusiform gyrus, right (R.) inferior parietal lobule, right (R.) inferior temporal gyrus, and right (R.) parahippocampal gyrus as well as the right (R.) precuneus (Table 4; Figure 3).

**Table 4:** Comparison of gray matter volume in significant regions between Aβ-positive and Aβ-negative groups (Mean ± SD).

Brain region	Aβ-positive (*n* = 30)	Aβ-negative (*n* = 20)	*F*	Raw *p* value	*q* value	Partial *η*^2^
L. fusiform gyrus	8447.50 ± 890.47	9622.50 ± 1825.30	7.436	0.009	0.047*	0.142
L. inferior parietal lobule	9927.20 ± 1637.62	12041.20 ± 1550.59	29.336	<0.001	0.003**	0.395
L. inferior temporal gyrus	9096.07 ± 1192.05	10610.35 ± 343.09	14.323	<0.001	0.003**	0.241
L. parahippocampal gyrus	1626.40 ± 343.09	2224.15 ± 343.09	29.737	<0.001	0.010*	0.398
L. posterior cingulate cortex	3198.97 ± 501.53	3799.95 ± 763.93	11.396	0.002	0.015*	0.202
L. precuneus	8443.43 ± 943.12	9581.45 ± 1041.33	14.169	<0.001	0.010*	0.239
R. caudal middle frontal gyrus	5130.63 ± 698.23	5759.45 ± 885.59	7.883	0.007	0.039*	0.149
R. entorhinal cortex	2076.57 ± 698.40	2769.80 ± 698.40	14.367	<0.001	0.010*	0.242
R. fusiform gyrus	8489.17 ± 1074.79	9951.70 ± 1684.14	9.902	0.003	0.020*	0.180
R. inferior parietal lobule	12523.10 ± 1074.79	15052.10 ± 1430.31	18.531	<0.001	0.010*	0.292
R. inferior temporal gyrus	8253.13 ± 1359.29	10976.30 ± 1561.59	15.468	<0.001	010*	0.256

Data are presented as mean ± standard deviation (SD). Intergroup volumetric comparisons were performed via analysis of covariance (ANCOVA), adjusted for age, sex, and estimated total intracranial volume (eTIV). Raw uncorrected *p* values were subjected to FDR multiple comparison correction to generate *q*-values, which serve as the sole metric to judge statistical significance throughout the manuscript. *q < 0.05, **q < 0.001.

**Figure 3:**
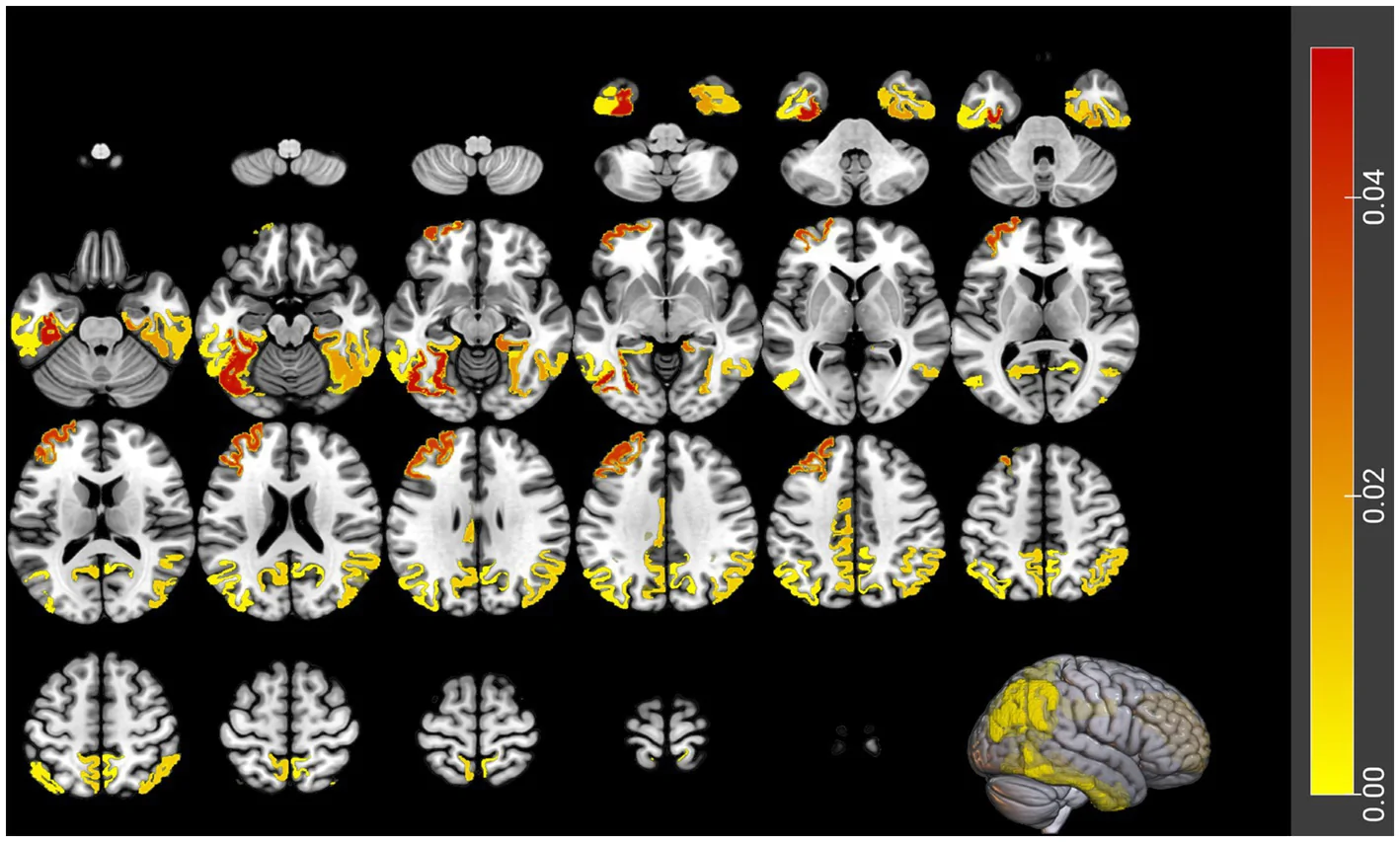
Brain regions with significant gray matter volume atrophy in Aβ-positive aMCI compared with Aβ-negative aMCI Voxel-based morphometry (VBM) analysis revealed significant gray matter volume atrophy in Aβ-positive aMCI patients relative to Aβ-negative aMCI patients, primarily in the bilateral fusiform gyrus, inferior parietal lobule, parahippocampal gyrus, precuneus, and right entorhinal cortex. The color bar represents the F-statistic values from the analysis of covariance (ANCOVA), with age, sex, and estimated total intracranial volume (eTIV) as covariates. All results were corrected for multiple comparisons using the false discovery rate (FDR) method (FDR-corrected *q* < 0.05).

### 3.5 Correlation analyses of multimodal biomarkers

#### 3.5.1 Correlations between electrophysiological metrics and cognitive performance

Partial correlation analyses controlling for age, gender and education were conducted to quantify associations between normalized scalp EEG spectral metrics and MMSE, MoCA cognitive scores stratified by participant subgroups. All raw uncorrected *p* values underwent FDR multiple comparison correction to generate q-values for significance judgment.

In the full combined cohort,the temporal scalp theta/alpha ratio showed a significant negative partial correlation with MMSE global scores (*r* = −0.474, 95%CI [−0.489, −0.121], raw *p* = 0.020, *q* = 0.026; Figure 4A). No significant partial correlations between any scalp EEG metric and MoCA total scores survived FDR correction (all *q* > 0.05; Figure 4B).

**Figure 4:**
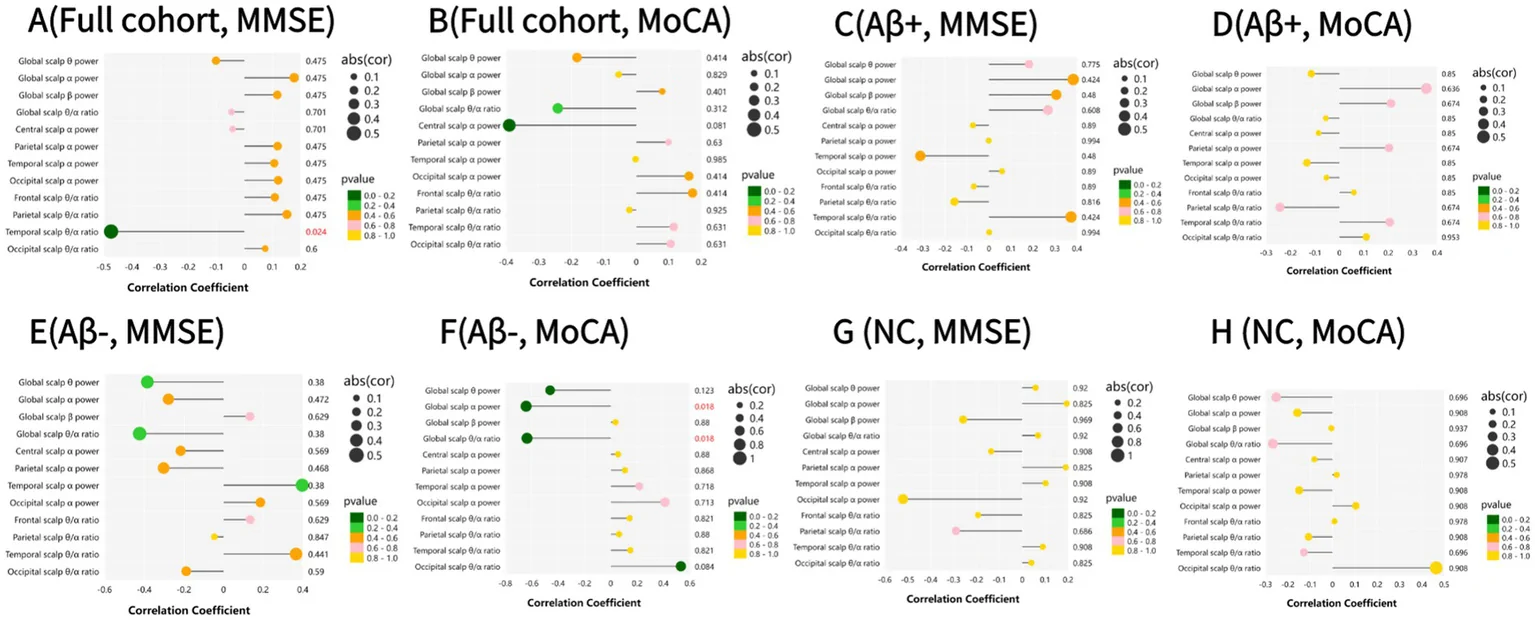
Partial correlations between scalp electrophysiological metrics and cognitive performance stratified by participant subgroups. **(A,B)** Associations between scalp EEG spectral metrics and MMSE **(A)**, MoCA **(B)** scores in the full combined cohort. **(C,D)** Associations between scalp EEG spectral metrics and MMSE **(C)**, MoCA **(D)** scores in the Aβ-positive aMCI subgroup. **(E,F)** Associations between scalp EEG spectral metrics and MMSE **(E)**, MoCA **(F)** scores in the Aβ-negative aMCI subgroup. **(G,H)** Associations between scalp EEG spectral metrics and MMSE **(G)**, MoCA **(H)** scores in the NC cohort. All correlation coefficients (*r*) were derived from partial correlation analyses adjusted for age, sex, and years of formal education. Error bars denote 95% confidence intervals (CI). Correlations reaching statistical significance after false discovery rate (FDR) multiple comparison correction (*q* < 0.05) are highlighted in colored markers.

Within the Aβ-positive aMCI subgroup, no statistically significant partial correlations between scalp EEG metrics and MMSE or MoCA scores were detected after FDR adjustment (all *q* > 0.05; Figures 4C,D).

For the Aβ-negative aMCI subgroup, global normalized scalp alpha power (*r* = −0.642, 95%CI [−0.809, −0.301], raw *p* = 0.002, *q* = 0.018) and global scalp theta/alpha ratio (*r* = −0.635, 95%CI [−0.804, −0.290], raw *p* = 0.003, *q* = 0.018) exhibited significant negative partial correlations with MoCA total cognitive scores (Figures 4E,F). No significant associations with MMSE scores were observed in this subgroup.

No significant partial correlations between scalp EEG spectral metrics and either cognitive scale were identified in the NC cohort (all *q* > 0.05; Figures 4G,H).

#### 3.5.2 Correlation between EEG metrics and Aβ burden

To explore the association between cortical oscillatory dysfunction and cerebral amyloid pathology, partial correlation analyses adjusted for age, gender and education were performed to quantify relationships between core normalized scalp EEG spectral metrics (parietal and temporal alpha relative power, temporal and parietal scalp theta/alpha ratio) and regional Aβ SUVR burden within predefined AD-vulnerable ROIs among the Aβ-positive aMCI subgroup.

The partial correlation analysis identified a significant positive association between the temporal scalp theta/alpha ratio and medial temporal lobe Aβ SUVR load in Aβ-positive aMCI participants (*r* = 0.501, 95%CI [0.168, 0.723], raw *p* = 0.005, *q* = 0.030; Figure 5). No statistically significant partial correlations survived FDR multiple comparison correction between the selected scalp EEG metrics and Aβ SUVR values in posterior cingulate/precuneus complex, lateral parietal cortex, lateral temporal cortex, global whole-cortex and medial prefrontal ROIs (all *q* > 0.05).

**Figure 5:**
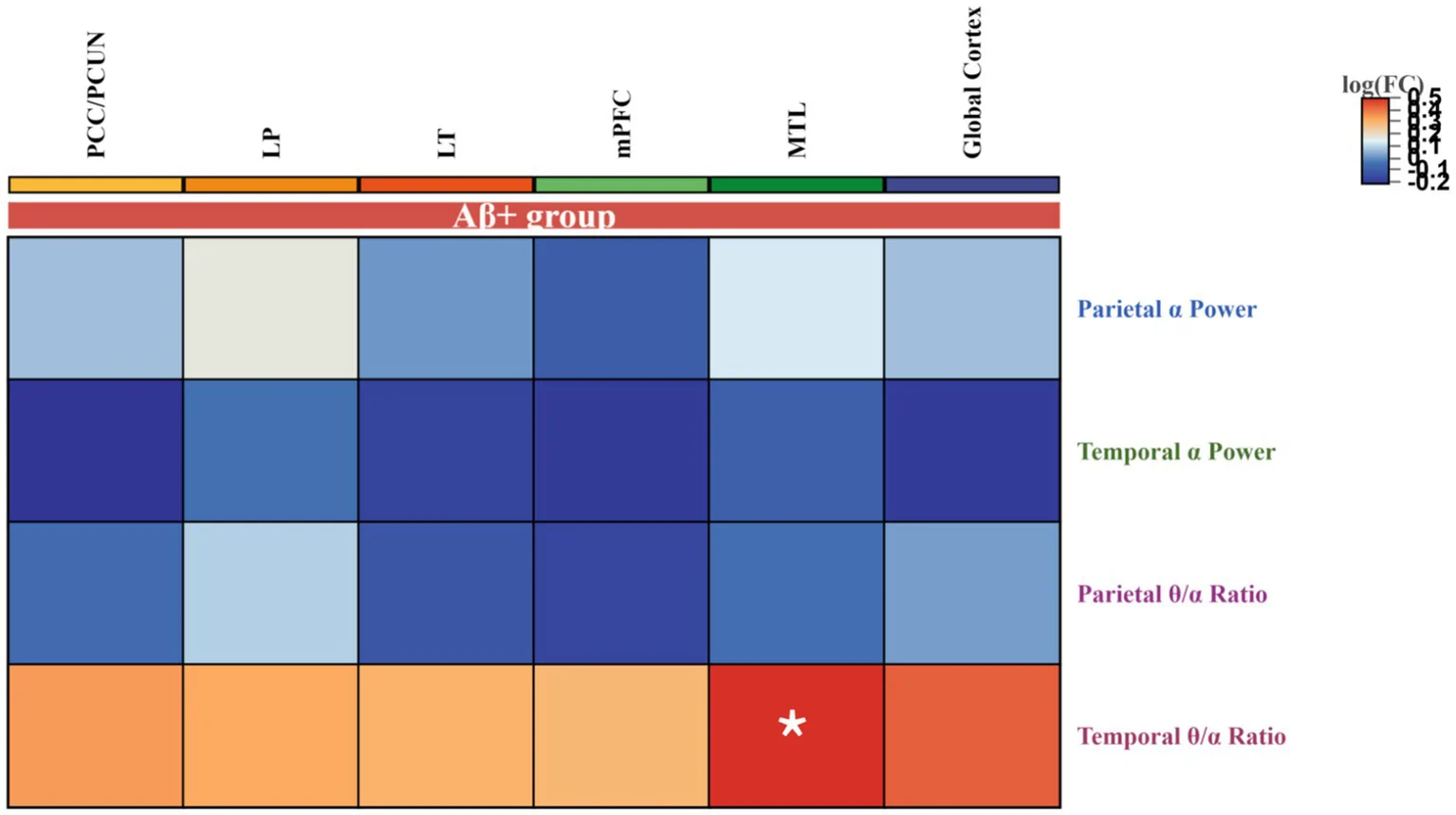
Partial correlations between core scalp EEG spectral metrics and regional Aβ SUVR burden in the Aβ-positive aMCI subgroup. This heatmap visualizes partial correlation coefficients between scalp EEG metrics (parietal α power, temporal α power, parietal θ/α ratio, temporal θ/α ratio) and Aβ deposition SUVR within established AD-vulnerable ROIs: posterior cingulate/precuneus complex (PCC/PCUN), lateral parietal cortex (LP), lateral temporal cortex (LT), medial prefrontal cortex (mPFC), medial temporal lobe (MTL), and global whole cortex. All correlation analyses were adjusted for age, sex, and years of formal education. The color scale corresponds to the magnitude of partial correlation coefficient (*r*); red tones denote positive correlations and blue tones denote negative correlations. The asterisk (*) marks the single significant positive association between temporal scalp θ/α ratio and medial temporal lobe Aβ SUVR (*r* = 0.501, FDR-adjusted *q* = 0.030).

#### 3.5.3 Association between Aβ burden, brain structure, and electrophysiology

To characterize the mutual interplay among cerebral amyloid pathology, regional gray matter volume and cortical oscillatory activity, stratified partial correlation analyses controlling for age, sex and estimated total intracranial volume (eTIV) were conducted separately within the Aβ-positive aMCI subgroup and Aβ-negative aMCI subgroup.

In the Aβ-positive aMCI subgroup, right precuneus gray matter volume exhibited significant negative partial correlations with Aβ SUVR burden across multiple AD-vulnerable ROIs: posterior cingulate/precuneus complex (*r* = −0.586, 95%CI [−0.772, −0.263], raw *p* = 0.001, *q* = 0.031), lateral parietal cortex (*r* = −0.527, 95%CI [−0.738, −0.181], raw *p* = 0.001, *q* = 0.037), global whole cortex (*r* = −0.538, 95%CI [−0.745, −0.196], raw *p* = 0.001, *q* = 0.037), and medial temporal lobe (*r* = −0.524, 95%CI [−0.736, −0.176], raw *p* = 0.001, *q* = 0.037). In addition, the temporal scalp theta/alpha ratio was positively associated with medial temporal lobe Aβ SUVR load (*r* = 0.511, 95%CI [0.161, 0.730], raw *p* = 0.006, *q* = 0.046; Figure 6). No other pairwise associations remained statistically significant following FDR multiple comparison correction.

**Figure 6:**
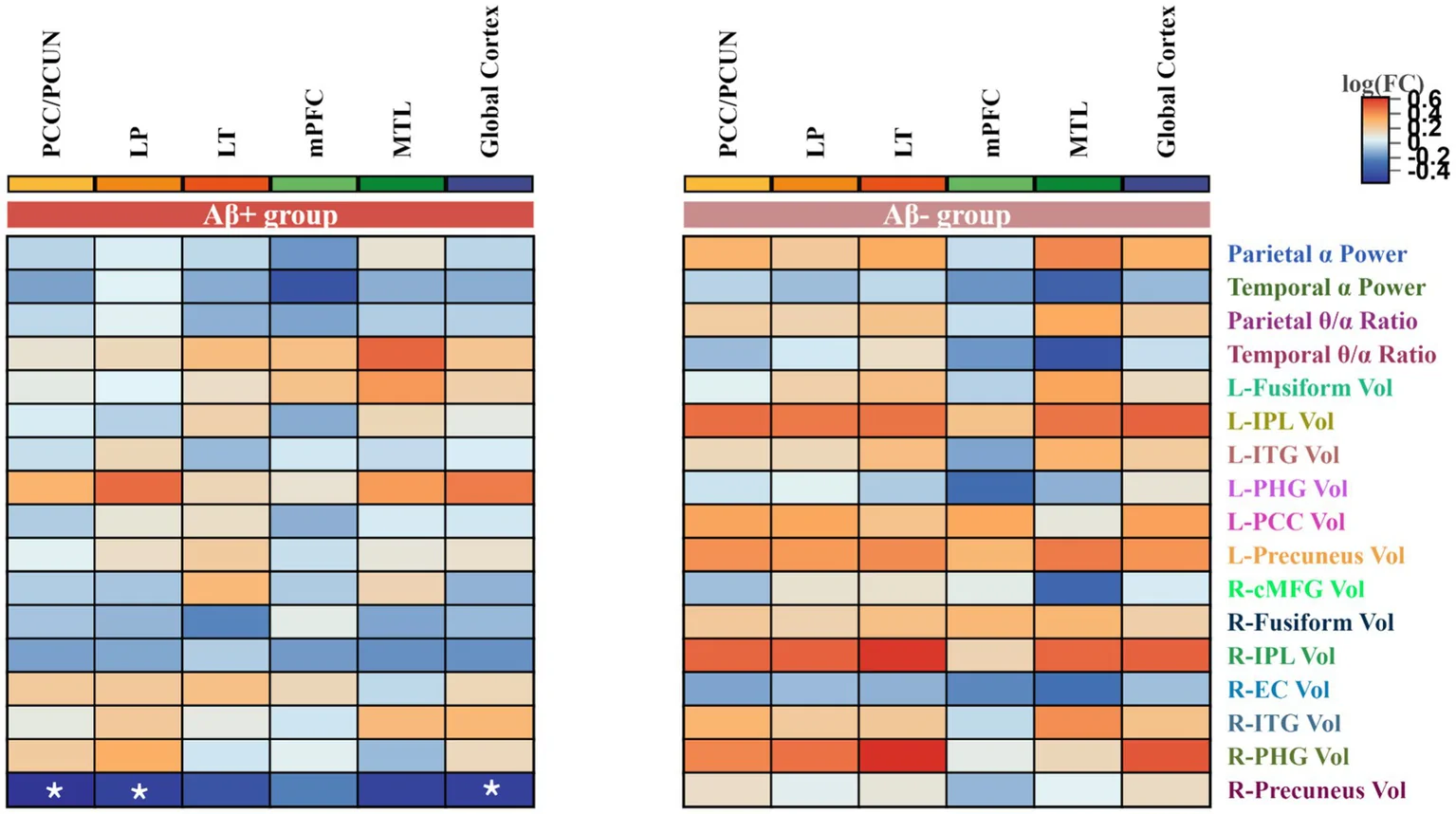
Partial correlation heatmaps depicting pairwise associations among regional Aβ SUVR burden, gray matter volume and scalp EEG spectral metrics in Aβ-positive and Aβ-negative aMCI subgroups. Left panel (Aβ-positive subgroup): Heatmap of partial correlation coefficients across Aβ burden ROIs, gray matter volumetric indices and scalp EEG metrics. Asterisks (*) mark pairwise associations with FDR-adjusted *q* < 0.05, encompassing negative correlations between right precuneus volume and multi-region Aβ deposition, as well as a positive correlation between temporal scalp θ/α ratio and medial temporal lobe SUVR. Right panel (Aβ-negative subgroup): No statistically significant partial correlations were detected after FDR multiple comparison correction, demonstrating the pathological specificity of the multimodal relationships observed in amyloid-positive participants. Color scale indicates the magnitude of partial correlation coefficient (*r*); all correlations were adjusted for age, sex and eTIV. Red shading represents positive correlations, and blue shading represents negative correlations. Abbreviations: PCC/PCUN, posterior cingulate/precuneus complex; LP, lateral parietal cortex; LT, lateral temporal cortex; mPFC, medial prefrontal cortex; MTL, medial temporal lobe; L., left; R., right; Vol, gray matter volume; θ/α, theta/alpha ratio; FDR, false discovery rate; eTIV, estimated total intracranial volume.

By contrast, no partial correlations reached FDR-adjusted significance thresholds in the Aβ-negative aMCI subgroup (all *q* > 0.05), supporting that the multimodal correlations identified above are specific to amyloid-positive pathological states.

### 3.6 Construction and evaluation of multimodal predictive model for Aβ-positive aMCI

#### 3.6.1 Screening of key biomarkers and construction of logistic regression model

Univariate intergroup comparison identified two variables showing significant differences between Aβ-positive and Aβ-negative aMCI subgroups: right precuneus gray matter volume (MRI volumetric metric) and temporal scalp θ/α ratio (EEG oscillatory metric). Both predictors were standardized via Z-score transformation and entered into the binary logistic regression model. One-thousand-sample bootstrap resampling was applied for internal model validation. Bootstrap-corrected results revealed that right precuneus volume (OR = 3.98, 95%CI: 1.39–11.37, raw *p* = 0.001) and temporal scalp θ/α ratio (OR = 5.41, 95%CI: 1.18–24.81, raw *p* = 0.030) were independent predictors for Aβ-positive pathological status (Table 5).

**Table 5:** Multivariate logistic regression analysis for predicting Aβ status (bootstrap-validated).

Variable	β	SE	Wald *χ*^2^	Raw *p*	OR	95% CI
R. precuneus volume	1.381	0.530	6.79	0.001*	3.98	1.39–11.37
Temporal θ/α ratio	1.689	1.555	4.71	0.030*	5.41	1.18–24.81
Constant	−0.519	0.538	0.93	0.335	0.60	—

*Raw *p* < 0.05 indicates a statistically significant difference.

#### 3.6.2 Diagnostic efficiency evaluation of multimodal model

ROC curve analysis with DeLong pairwise comparison was performed to compare discriminative performance across single-modal biomarkers and the combined multimodal classifier. The multimodal auxiliary classification model integrating right precuneus gray matter volume and temporal scalp θ/α ratio achieved favorable discriminative capacity for separating Aβ-positive aMCI from Aβ-negative aMCI (AUC = 0.852, 95%CI: 0.748–0.955, raw *p* < 0.001). DeLong test confirmed its AUC was significantly higher than either single-modal biomarker alone: temporal scalp θ/α ratio (AUC = 0.822) and right precuneus volume (AUC = 0.778) (Table 6; Figure 7A).

**Table 6:** AUC comparison of different models for discriminating Aβ status.

Model	AUC	SE	Raw *p*	95% CI
Multimodal combined model	0.852	0.053	<0.001*	0.748–0.955
Temporal scalp θ/α ratio only	0.822	0.059	<0.001*	0.706–0.937
R. precuneus volume only	0.778	0.065	0.001*	0.650–0.907

All *p* values listed are uncorrected raw *p* values generated from ROC analysis. *Raw *p* < 0.05 represents statistically meaningful discriminative power for each individual model. Pairwise AUC differences between the multimodal model and single-modal biomarkers were formally tested via the DeLong test. This model is only suitable for auxiliary Aβ pathological stratification within this single-center cohort and lacks external validation data to support generalized clinical application.

**Figure 7:**
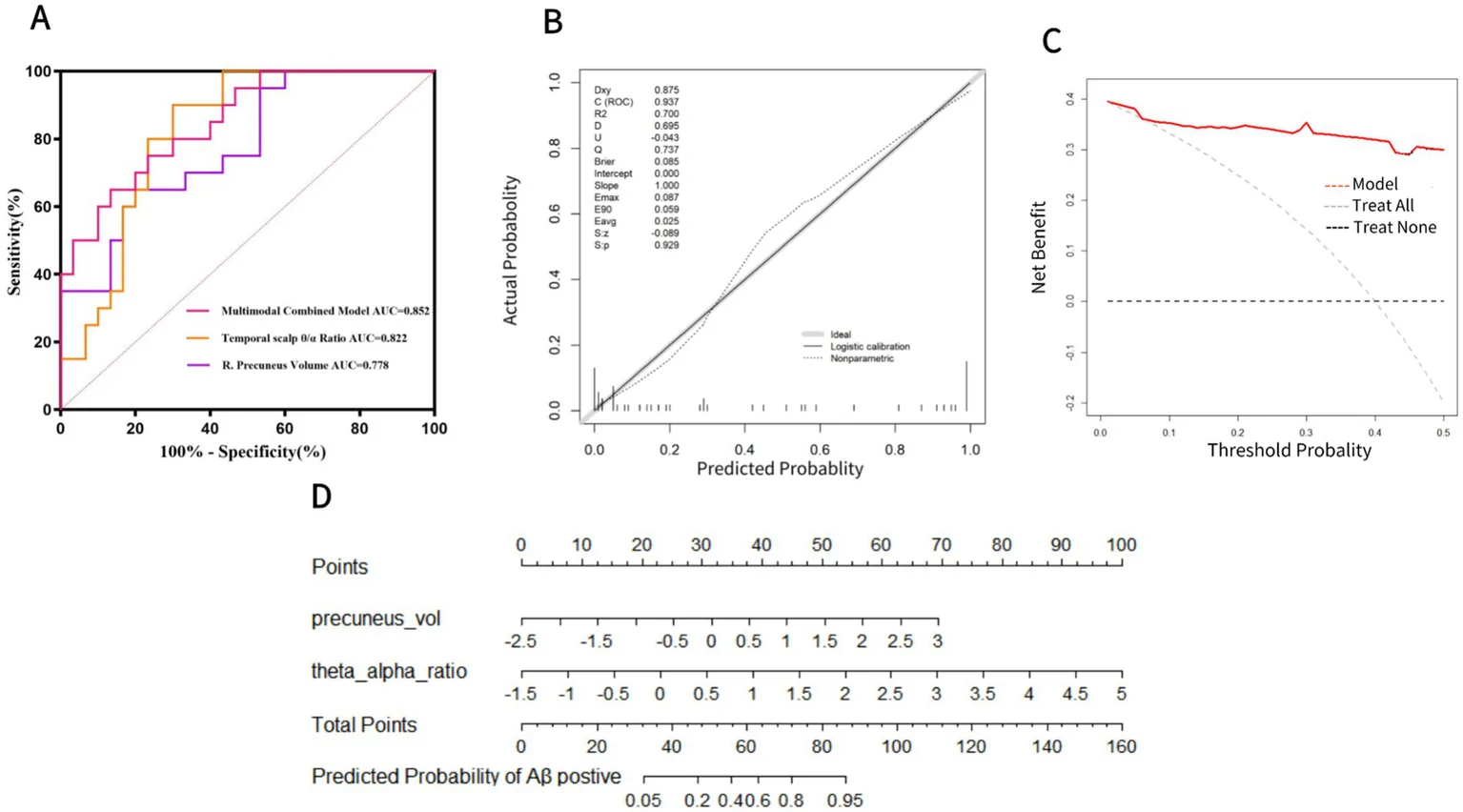
Establishment and performance evaluation of the multimodal auxiliary classification model for Aβ pathological stratification in aMCI subgroups **(A)** Receiver operating characteristic (ROC) curves comparing discriminative performance of three classifiers for separating Aβ-positive aMCI from Aβ-negative aMCI: combined multimodal model, temporal scalp θ/α ratio single metric, and right precuneus gray matter volume single metric. DeLong test was used for pairwise AUC comparison. **(B)** Calibration curve of the multimodal auxiliary model, illustrating favorable consistency between model-predicted Aβ-positive probabilities and real observed pathological status within this cohort. **(C)** Decision curve analysis (DCA) quantifying standardized clinical net benefit of the multimodal classifier across different risk threshold probabilities. **(D)** Clinical nomogram integrating right precuneus gray matter volume and temporal scalp θ/α ratio to generate individualized Aβ-positive risk estimates for auxiliary pathological stratification.

#### 3.6.3 Model calibration and clinical utility analysis

Calibration curve assessment demonstrated favorable internal calibration performance for this multimodal auxiliary classification model (Figure 7B). Bootstrap-corrected model metrics were as follows: C-index = 0.84 (95%CI: 0.75–0.95), calibration intercept = −0.05 (95%CI: −0.94–1.00), calibration slope = 0.89 (95%CI: 0.29–1.55). These metrics reflect strong consistency between model-predicted Aβ-positive probabilities and observed true pathological status within this cohort.

Decision curve analysis (DCA) quantified standardized clinical net benefit across a full range of risk thresholds. Within the threshold probability range of 10–40%, the multimodal combined classifier maintained a persistently higher net benefit curve relative to the two reference strategies (assume all participants are Aβ-positive / assume no participants are Aβ-positive) (Figure 7C), revealing favorable discriminative value for auxiliary pathological stratification in this single-center cohort.

A clinical nomogram was constructed based on the final joint prediction model (Figure 7D), which integrated two predictive factors: right precuneus volume and temporal θ/α ratio. The individualized Aβ-positive prediction probability can be conveniently queried by mapping the individual indicator values to scores and summing them up.

## 4 Discussion

This study systematically investigated resting-state EEG spectral characteristics and gray matter volume alterations among aMCI participants stratified by cerebral Aβ deposition status, integrating neuroelectrophysiology, structural/molecular imaging and standardized neuropsychological assessments. We further quantified pairwise correlations between multimodal neural biomarkers, cerebral Aβ burden, and domain-specific cognitive performance. Collectively, our core results reveal that Aβ-positive aMCI individuals exhibit distinct scalp EEG oscillatory dysfunction and regional gray matter atrophy within canonical AD-vulnerable brain networks. The temporal scalp θ/α ratio and right precuneus gray matter volume act as promising non-invasive auxiliary biomarkers to distinguish Aβ-positive aMCI from Aβ-negative aMCI subgroups (Rodriguez et al., 2026). Furthermore, the multimodal auxiliary classification model combining these two indicators yields significantly superior discriminative capacity compared with single-modal biomarkers, as formally validated via DeLong test. Importantly, all detected structural and electrophysiological aberrations correlate with cerebral Aβ pathology; nevertheless, such neural impairments cannot be solely attributed to Aβ plaque deposition, given the unmeasured confounding effect of medial temporal tau neurofibrillary pathology within this single-center cohort (Papp et al., 2017).

### 4.1 Distinct resting-state scalp EEG spectral patterns characterize Aβ-positive aMCI

In the present cohort, the Aβ-positive aMCI subgroup displayed consistent global scalp EEG abnormalities, including elevated theta relative power, attenuated alpha and beta relative power, and an increased scalp θ/α ratio, with the most severe oscillatory impairment localized to temporal, parietal and occipital scalp electrode clusters. By contrast, no statistically significant spectral differences were identified between the Aβ-negative aMCI group and cognitively normal controls after FDR multiple comparison correction. This observation confirms that quantitative scalp EEG oscillatory abnormalities in aMCI are tightly coupled to cerebral Aβ pathological burden, rather than non-specific age-related electrophysiological shifts. Consistent with previous longitudinal cohort data (Zhou et al., 2024), cortical alpha rhythm suppression and theta slow-wave augmentation in Aβ-positive aMCI reflect disrupted cortical neuronal synchronization and impaired cholinergic neuromodulation. Specifically, alpha oscillations dominate spontaneous cortical activity during wakeful rest, and their attenuation indicates compromised synchronous neuronal firing capacity. Exaggerated theta activity is well-established as a marker of disrupted default mode network (DMN) function and medial temporal mnestic circuit dysfunction, which correlates robustly with episodic memory decline (Gonzalez-Escamilla et al., 2015).

As a composite quantitative electrophysiological metric that indexes cortical oscillatory balance, the scalp θ/α ratio sensitively captures early functional neural damage across the AD pathological continuum, and its progressive elevation parallels worsening global cognitive performance (Sarazin et al., 2010). Our ROC analysis with DeLong pairwise comparison confirmed that the temporal scalp θ/α ratio achieved favorable discriminative capacity for separating Aβ-positive from Aβ-negative aMCI (AUC = 0.875), consistent with prior published work by Kim et al. (2021). Pathologically, these findings revise the simplified model of regional pathological onset in preclinical AD: insoluble Aβ plaques initially accumulate diffusely across neocortical and DMN hubs, whereas tau neurofibrillary tangles first develop within medial temporal structures including the hippocampus and entorhinal cortex (Jack et al., 2013). Accordingly, the temporal scalp slow-wave abnormalities observed herein likely reflect synergistic neural dysfunction induced by local neocortical Aβ deposition and coexisting unmeasured medial temporal tau pathology, rather than Aβ-mediated neuronal injury in isolation (García-Carlos et al., 2024).

In addition, conventional qualitative visual EEG grading failed to detect intergroup spectral differences across the three study cohorts, highlighting the inherent limitations of subjective visual EEG interpretation for early preclinical AD screening. Macroscopic visual assessment only identifies severe, overt disruptions of background EEG activity, while subtle, distributed frequency-band shifts at the aMCI prodromal stage can only be reliably quantified via spectral decomposition analysis (McKay et al., 2025). The present study further demonstrates that early AD-related neural dysfunction manifests as continuous quantitative gradients of oscillatory power, rather than discrete qualitative waveform abnormalities, supporting the translational application of quantitative resting-state scalp EEG for pathological subtyping of aMCI.

### 4.2 Region-specific gray matter atrophy within AD-associated networks in Aβ-positive aMCI

Voxel-based morphometry revealed significant gray matter volume loss across 13 predefined AD-vulnerable brain regions in Aβ-positive aMCI participants, encompassing bilateral fusiform gyrus, inferior parietal lobule, parahippocampal gyrus, precuneus, and the right entorhinal cortex. These anatomical regions constitute core functional hubs of the DMN, medial temporal episodic memory circuit, and temporoparietal association cortex, which are selectively vulnerable to AD pathological cascades throughout disease progression (Papp et al., 2017). The medial temporal lobe is critical for episodic memory consolidation, and its volumetric atrophy correlates closely with amnestic cognitive impairment at the symptomatic prodromal stage (Sarazin et al., 2010). As a central posterior hub of the DMN, the precuneus mediates memory retrieval and high-order executive processing; its gray matter loss correlates with elevated cortical Aβ burden and global cognitive deterioration (Fischer et al., 2025).

Within this cohort, right precuneus gray matter atrophy exhibited the largest effect size and independent auxiliary discriminative value for identifying Aβ-positive aMCI (AUC = 0.778), consistent with results reported by Stricker et al. (2019). Widespread temporoparietal association cortex atrophy detected in Aβ-positive aMCI may reflect trans-synaptic propagation of mixed Aβ-tau pathology across interconnected neural circuits (Vonk et al., 2020). Collectively, this targeted pattern of gray matter atrophy confirms that Aβ-positive aMCI represents the early symptomatic phase of the AD pathological continuum. Nevertheless, aligned with contemporary neuropathological evidence, medial temporal gray matter loss observed in our sample is primarily driven by tau neurofibrillary degeneration, while Aβ plaque deposition acts as a synergistic pathological accelerator that exacerbates irreversible neuronal atrophy (Dautricourt et al., 2021).

### 4.3 Multimodal correlations among neural biomarkers, Aβ burden and cognitive performance

Partial correlation analyses adjusted for age, sex and eTIV demonstrated that elevated temporal scalp θ/α ratio was positively associated with medial temporal Aβ SUVR load in Aβ-positive aMCI participants, while reduced right precuneus gray matter volume correlated with higher Aβ deposition across the posterior cingulate-precuneus complex, lateral parietal cortex, medial temporal lobe and global neocortex. These pairwise associations partially support the classic Aβ cascade hypothesis, yet cannot establish direct causal relationships between Aβ accumulation and subsequent neural damage (Livingston et al., 2024). Mechanistically, medial temporal neurodegeneration in AD arises from synergistic interactions between Aβ plaque aggregation, chronic neuroinflammation, secondary tau hyperphosphorylation and interregional tau propagation. Aβ deposition remodels the perineuronal microenvironment to facilitate tau tangle formation, progressive neuronal loss and inter-regional circuit desynchronization, ultimately driving temporal scalp oscillatory slowing and focal gray matter shrinkage (Das et al., 2019). Similarly, elevated precuneus Aβ burden exacerbates permanent neuronal loss and DMN functional disruption in conjunction with coexisting tau pathology (Inagawa et al., 2026). In summary, scalp EEG and structural MRI biomarkers reflect combined functional-structural neurodegeneration induced by mixed Aβ-tau pathological burden, rather than amyloid-specific damage alone.

Regarding cognitive correlates, the temporal scalp θ/α ratio exhibited a significant negative partial correlation with global MMSE scores across all participants, and left parahippocampal gyrus gray matter volume correlated positively with MoCA memory subscores within the Aβ-positive subgroup. These associations validate that quantitative oscillatory and morphological abnormalities track domain-specific cognitive deterioration in AD-spectrum aMCI (Tripanpitak et al., 2026). Notably, divergent correlation patterns emerged in the Aβ-negative aMCI cohort: global scalp alpha relative power and scalp θ/α ratio both displayed significant negative partial correlations with total MoCA scores. This distinct association profile further corroborates the substantial pathological heterogeneity of aMCI, where cognitive decline among Aβ-negative individuals is predominantly driven by non-AD etiologies including cerebral small vessel disease, vascular brain injury and α-synuclein Lewy body pathology (Malotaux et al., 2024). This key finding underscores the clinical necessity of Aβ-status stratification to enable targeted pathological evaluation and personalized intervention.

### 4.4 Multimodal fusion optimizes auxiliary pathological stratification of Aβ-positive aMCI

Single-modal biomarkers carry inherent limitations for discriminating Aβ-positive aMCI from non-AD aMCI variants. In the current study, the binary logistic regression auxiliary classification model integrating the temporal scalp θ/α ratio (functional scalp EEG biomarker) and right precuneus gray matter volume (structural sMRI biomarker) achieved significantly superior discriminative performance (AUC = 0.852) relative to either single indicator alone, as verified via DeLong pairwise AUC comparison, confirming the complementary predictive value of multimodal data fusion (Kang et al., 2021). Scalp resting-state EEG captures millisecond-timescale synchronous cortical oscillatory activity, reflecting reversible early functional neural disturbance; by contrast, sMRI-derived gray matter volume quantifies permanent macroscopic neuronal morphological loss (Bae et al., 2023). The two imaging modalities index AD-spectrum neurodegeneration across independent functional and structural dimensions, supplying non-overlapping predictive information for Aβ pathological stratification. Multivariate bootstrap-validated logistic regression further confirmed both biomarkers as independent predictors of Aβ-positive pathological status.

In addition, the constructed clinical nomogram exhibited favorable internal calibration and standardized clinical net benefit across broad risk threshold ranges, supporting its utility for individualized Aβ-positive risk estimation as an auxiliary screening tool. Currently, ^18^F-labeled Aβ PET remains the clinical reference standard for cerebral amyloid quantification, yet its high financial cost and ionizing radiation exposure preclude widespread population-level screening and serial longitudinal follow-up (Kim et al., 2024). The EEG-sMRI multimodal classifier developed herein features full non-invasiveness, low economic burden and high test–retest repeatability, which can serve as a cost-effective preliminary screening pipeline to triage high-risk candidates for definitive clinical Aβ PET scanning and stratified therapeutic intervention. Critically, this model is exclusively intended for auxiliary pathological subtyping within the AD continuum; it cannot independently confirm a clinical diagnosis of AD nor predict longitudinal progression to dementia.

### 4.5 Limitations and future perspectives

Several key limitations of this study must be explicitly acknowledged. First, this cross-sectional single-time-point design only enables detection of correlational associations between multimodal biomarkers and pathological phenotypes, and cannot resolve causal relationships or track dynamic longitudinal shifts of neural biomarkers alongside progressive disease (Galluzzi et al., 2016). Large-scale multicenter longitudinal cohort studies are required to characterize biomarker evolutionary trajectories and validate their prognostic capacity for subsequent cognitive decline.

Second, rigid imaging-based aMCI inclusion criteria introduce methodological bias including analytical circularity and restricted generalizability of our findings. All enrolled aMCI participants were required to satisfy the hippocampal amnesic phenotype with definitive baseline medial temporal atrophy visible on sMRI prior to Aβ PET stratification. That is, medial temporal gray matter atrophy served simultaneously as an enrollment screening threshold and the primary structural outcome biomarker, which may artificially amplify volumetric differences between Aβ-positive and Aβ-negative subgroups. Furthermore, this inclusion rule excludes aMCI cases with intact medial temporal morphology driven by vascular cognitive impairment, dementia with Lewy bodies and other non-AD pathologies. Accordingly, our multimodal biomarkers and predictive model are only generalizable to aMCI patients presenting baseline medial temporal atrophy, and cannot be extended to the full heterogeneous aMCI population. Future research adopting relaxed imaging entry criteria is required to validate the external reproducibility of our results.

Third, the absence of tau-specific molecular imaging (tau PET) represents a major interpretative limitation. Accumulated high-level pathological evidence demonstrates that medial temporal tau neurofibrillary tangles, rather than Aβ plaques, are the primary driver of cortical oscillatory slowing and focal neuronal atrophy in prodromal AD (Almkvist and Winblad, 1999). Without quantitative tau load measurements, we cannot disentangle the independent pathological contributions of Aβ and tau to the scalp EEG and structural MRI alterations identified herein; all pathological interpretations within this manuscript address the combined effects of mixed AD co-pathologies.

Fourth, the 21-channel scalp EEG system used in this study possesses limited spatial resolution, and surface scalp oscillatory signals cannot precisely localize electrical activity originating from deep limbic brain structures. Future work incorporating high-density EEG paired with source reconstruction algorithms will improve anatomical precision of electrophysiological biomarkers.

Fifth, the present analysis only extracted gray matter volumetric features, omitting assessments of white matter microstructural integrity and whole-brain resting-state functional connectivity (Vanneste et al., 2021). Integration of multi-dimensional MRI quantitative metrics may further enhance the discriminative performance of multimodal classification models.

Future investigative directions are outlined as follows: (1) Establish multicenter, large-sample longitudinal follow-up cohorts to validate the prognostic value of combined scalp EEG-sMRI biomarkers for aMCI disease progression; (2) Integrate tau PET, peripheral blood AD biomarkers and multi-modal molecular imaging to disentangle the independent pathological effects of Aβ and tau on neural dysfunction; (3) Deploy machine learning pipelines to extract high-dimensional quantitative EEG and MRI features to build more accurate pathological classification models; (4) Conduct prospective clinical intervention trials to evaluate whether optimized multimodal biomarker panels can quantify therapeutic response for AD-spectrum cognitive impairment (Moretti et al., 2013).

## 5 Conclusion

Aβ-positive aMCI participants display characteristic quantitative resting-state scalp EEG spectral abnormalities (elevated theta relative power, reduced alpha/beta relative power, increased temporal scalp θ/α ratio) and focal gray matter atrophy across DMN and medial temporal mnestic brain hubs, and these combined functional-structural neurodegenerative alterations correlate significantly with cerebral Aβ burden and domain-specific cognitive impairment. The temporal scalp θ/α ratio and right precuneus gray matter volume represent reliable non-invasive auxiliary biomarkers for identifying Aβ-positive aMCI. The multimodal auxiliary classification model integrating these two indicators achieves statistically superior discriminative capacity relative to single-modal biomarkers, with favorable translational potential for individualized amyloid risk stratification. Collectively, this multimodal strategy delivers a low-cost, radiation-free auxiliary pipeline for early pathological subtyping within the AD continuum. Importantly, this study clarifies that all observed neural abnormalities arise from synergistic mixed Aβ and unmeasured tau pathological effects; strict cohort inclusion criteria and the absence of tau PET biomarkers limit the broad generalizability and causal mechanistic interpretation of our findings (Ye et al., 2014).

## Data Availability

The raw data supporting the conclusions of this article will be made available by the authors, without undue reservation.

## Glossary

aMCI: amnestic mild cognitive impairment
AD: Alzheimer’s disease
ADL: Activities of Daily Living Scale
Aβ: β-amyloid
ANCOVA: analysis of covariance
AUC: area under the curve
BMI: body mass index
BAPL: Brain Amyloid Plaque Load
CAR: common average reference
CI: confidence interval
CDR: Clinical Dementia Rating
DK: atlas Desikan-Killiany atlas
DCA: decision curve analysis
DMN: default mode network
EEG: electroencephalogram
FDG: fluorodeoxyglucose
FFT: fast Fourier transform
FDR: false discovery rate
HAM-A: Hamilton Anxiety Scale
HAMD: Hamilton Depression Scale
HIS: Hachinski Ischemic Score
MMSE: Mini-Mental State Examination
MoCA: Montreal Cognitive Assessment
MRI: magnetic resonance imaging
NC: normal controls
OSEM: ordered subset expectation maximization
PET: positron emission tomography
ROI: region of interest
ROC: receiver operating characteristic
SD: standard deviation
SUVR: standardized uptake value ratio
TOF: time of flight
VBM: voxel-based morphometry
eTIV: estimated total intracranial volume
